# *Lentinula edodes* Mycelium as Effective Agent for Piroxicam Mycoremediation

**DOI:** 10.3389/fmicb.2019.00313

**Published:** 2019-02-21

**Authors:** Bożena Muszyńska, Monika Dąbrowska, Małgorzata Starek, Paweł Żmudzki, Jan Lazur, Jolanta Pytko-Polończyk, Włodzimierz Opoka

**Affiliations:** ^1^Department of Pharmaceutical Botany, Faculty of Pharmacy, Jagiellonian University Medical College, Kraków, Poland; ^2^Department of Inorganic Chemistry, Faculty of Pharmacy, Jagiellonian University Medical College, Kraków, Poland; ^3^Department of Medicinal Chemistry, Faculty of Pharmacy, Jagiellonian University Medical College, Kraków, Poland; ^4^Department of Integrated Dentistry, Jagiellonian University Medical College, Kraków, Poland

**Keywords:** bioremediation, edible mushroom, *Lentinula edodes*, piroxicam, UPLC/MS/MS analysis

## Abstract

Pollution of the environment with inorganic and organic substances is one of the main problems in the world. For this reason, it is necessary to conduct researches for effective methods of biodegradation of xenobiotics, including drugs whose unmetabolized forms are introduced into the environment, especially into water. One possible solution to this problem may be the use of white rot fungi, such as *Lentinula edodes*. This is an edible species used in medicine because of its beneficial anti-cancer, hypocholesterolemic, hypotensive, hypoglycemic and antioxidant effects. Due to the fact that the mycelium of *L. edodes* produces enzymes with oxidizing properties that can degrade xenobiotics. The aim of the work was verification if *in vitro* cultures of *L. edodes* can be used for bioremediation of non-steroidal, anti-inflammatory drug: piroxicam. For this purpose, the *in vitro* culture of *L. edodes* was derived and the mycelial cultures of this species enriched with piroxicam were analyzed. The biodegradation pathway of piroxicam by *L. edodes* mycelium was carried out by the UPLC/MS/MS method. The degradation process of piroxicam was found to affect primarily the linker between the thiazine and the piperidine ring, leading to its oxidation and cleavage. Additionally, oxidation of the benzothiazine moiety was observed, leading to hydroxylation and oxidation of the phenyl ring as well as oxidation of the thiazine ring leading to partial or complete removal of the sulfonamide moiety. It seems that the degradation process led finally to 2-hydroxybenozquinone, which may be further oxidized to inorganic compounds. What’s more, concentration of piroxicam in *in vitro* cultures of *L. edodes* was not correlated with effectiveness of biodegradation of this compound – on each experimental series, the level of degradation was the same. The results confirm the possibility of using the investigated *L. edodes* mycelium for remediation of piroxicam.

## Introduction

Pollution of the environment with inorganic and organic substances has become one of the main problems in the world. The main goal is to look for an effective method of biodegradation of xenobiotics, including drugs whose excess or unmetabolized forms are introduced into the environment (Das, 2005; Kulshreshtha et al., 2014; Kryczyk et al., 2017).

Pharmaceuticals have an important role in the treatment and prevention of disease in humans and animals. Unfortunately, they can also have unintended effects on living organisms in the environment. If possible, side effects on human and animal health are usually investigated, the study of potential environmental impact has only recently become a subject of interest for researchers. However, metabolism products or the combination of various biologically active compounds may have unpredictable effects.

The main source of contamination of water or soil with medicines are excrements. After being transported to municipal sewage systems, they reach the environment. Active substances may appear unchanged or in the form of metabolites. Wastewater treatment plants essentially remove solids and organic substances, while recalcitrant pollutants are often neglected.

Studies conducted all over the world indicate that NSAIDs are present in seawater, surface water and sewage (Lolic et al., 2015; Mainero Rocca et al., 2015). The presence of such drugs, even at low concentrations, can have a significant impact on water and land systems (Bácsi et al., 2016).

Piroxicam is a non-steroidal anti-inflammatory agent, widely used for chronic inflammatory conditions, particularly in treatment of rheumatoid arthritis, osteoarthritis, ankylosing spondylitis, pain in musculoskeletal disorders, acute gout and postoperative and postpartum pain. It characterized by low solubility and high permeability, therefore, adverse effects were reported when large doses are taken over a long period of time (Saganuwan and Orinya, 2016).

Piroxicam is a drug which mechanism of action is non-selective inhibition of cyclooxygenases. The ease with which a piroxicam molecule can transform from one prototropic form to another, depending on the environment conditions. This can be associated with the presence of hydrophobic chains with different lengths and surface charges in the environment which affects the interaction of piroxicam with micelles. Some studies show the impact of these factors on determining this balance (Chakraborty and Sarkar, 2005).

In the available literature there are works on the stability of piroxicam under different reaction environment conditions (Lemp et al., 2001; Puthli and Vavia, 2009; Starek et al., 2009; Aminuddin et al., 2011; Modhave et al., 2011). They indicate significant degradation of the molecule under hydrolytic, oxidative and photo-neutral conditions, and at the same time stability in dry heat and exposure to light in the solid state. The advanced degradation of piroxicam by oxidation (ozone or H_2_O_2_/O_3_ treatment) has been rarely explored (Feng et al., 2015). Lianou et al. (2018) presented a sonochemical oxidation of piroxicam with proposition of a degradation pathways. Authors point to ultrasonic irradiation as a promising technology with relatively high efficiency, but the scale of the process should still be analyzed in order to be able to be used commercially.

Some studies of the level of pharmaceuticals in wastewater from treatment, including many NSAIDs such as ibuprofen and naproxen, may lead to the finding of their toxic concentration in relation to fishes (Qun et al., 2008). Prolonged exposure of fish to environmentally harmful concentrations of drugs may bioaccumulate and deteriorate their health (Schwaiger et al., 2004). It shows how important research is on the possibilities of eliminating pollutants from the environment. In some cases, it turned out that metabolites are more toxic than the original compound, which is an additional problem in remediation (van Leeuwen et al., 2011).

The natural ability of mushroom to neutralize toxic substances from their environment encouraged scientists to evaluate their use in biodegradation of xenobiotics in *in vitro* conditions. Biodegradation ability is connected with synthesis of non-selective enzymes in hyphae, especially the ones which can be excreted extracellularly and high efficiency in environmental cleansing processes is also caused by rapid growth, production of large amounts of biomass and widespread occurrence of hyphae in the environment (Ashoka et al., 2002; Ma and Zhai, 2014). The processes of mycoremediation use different mechanisms than those found in bacteria. The main advantage is that they do not require initial preparation for specific contamination (Asamudo et al., 2005). Lang describes that white rot fungi (WRF) show remarkable abilities to transform resistant impurities such as polycyclic aromatic hydrocarbons to non-toxic products (Lang et al., 1995). The main mechanism of degradation catalyzed by white rot fungi concerns the degradation of lignin in the natural environment. Extracellular lignin modifying enzymes (LMEs) have low substrate specificity, so they are capable of mineralizing a large number of various highly resistant organic impurities structurally related to lignin (Novotný et al., 2004; Kaneda et al., 2008; Wong, 2009).

Thanks to this ability, it is possible to use mushroom cultures to remove organic pollutants from the environment. For this reason, the edible mushroom *L. edodes* belonging to the WRF species was selected in this study. *L. edodes* is intensively studied due to the presence of compounds with therapeutic effects such as anti-cancer, hypocholesterolemic, hypotensive, hypoglycemic, antioxidant antifungal and antibacterial in fruiting bodies (Casaril et al., 2011; Mleczek et al., 2017; Muszyńska et al., 2017). Some of these compounds are well characterized and used, e.g., lentinan, lentinacin, eritadenin. The most studied active substance isolated from *L. edodes* is pure β-(1,3)-D-glucan known as lentinan. As a result of *in vitro* and *in vivo* studies, the extracts of *L. edodes* and lentinan have been shown to have antitumor activity and for this reason preparations that contain them are used in conventional oncological therapy (Fulushima et al., 2001).

Due to the fact that mycelium *L. edodes* produces enzymes with oxidizing properties that can degrade xenobiotics, the aim of the work was to use this edible species for the human body and the environment to remedy them from the commonly used non-steroidal anti-inflammatory drug piroxicam. For this purpose, the *in vitro* culture of *L. edodes* was derived, followed by shaking mycelial cultures of this species to which piroxicam was added. The piroxicam biodegradation pathway was analyzed by UPLC/MS/MS.

## Materials and Methods

### Reagents

The chemicals: glucose, maltose extract, casein hydrolyzate, L-asparagine, adenine, B_1_ and B_6_ vitamins, agar, yeast extract were purchased from Sigma-Aldrich (St. Louis, MO, United States). NH_4_Cl, KH_2_PO_4_, MgSO_4_⋅7H_2_O, CaCl_2_⋅6H_2_O, FeCl_3_, MnSO_4_⋅H_2_O, ZnSO_4_⋅7H_2_O were bought from PPHGolpharm (Kraków, Poland). HPLC grade methanol and dichloromethane came from Merck (Darmstadt, Germany). Water (quadruple-distilled) with a conductivity of less than 1 μS cm^-1^ was obtained using an S2-97A2 distillation apparatus (ChemLand, Stargard Szczeciński, Poland). Reference substance of piroxicam was from FIS (Vicenza, Italy).

### Mushroom Material

The fruiting bodies of *L. edodes* (Berk.) Pegler of commercial origin, purchased at a supermarket in Poland. Taxonomic identification was based on the MycoKey 4.1^1^ by Muszyńska. Representative samples of the material are kept at the Department of Pharmaceutical Botany UJ CM (Kraków, Poland). Some of the young sporocarps of *L. edodes* were used to develop *in vitro* culture. Fragments of the hymenial part of sporocarps with an area of approx. 2 mm^2^ were used. The explants were degreased with 70% alcohol. After several rinsing with sterile redistilled water, the sporocarps fragments were transferred to the BD Sabouraud Agar with chloramphenicol (laminar airflow) medium. The solid cultures were incubated in a thermostat (ST500/B/40 Pol-Eko-Apparatus) at 23°C for 2 weeks (Picture 1). Microscopic analysis of obtained mycelium of *L. edodes* was carried out by Muszyńska. It showed a homogeneous nature of hyphae, free from contamination with other strains of fungi or bacteria. In the microscopic image, numerous branches and transverse walls were visible as well as the dikaryotic phase. Representative petri plates with mushroom material named LES/KB2 are kept at the Department of Pharmaceutical Botany UJ CM (Kraków, Poland).

**PICTURE 1:**
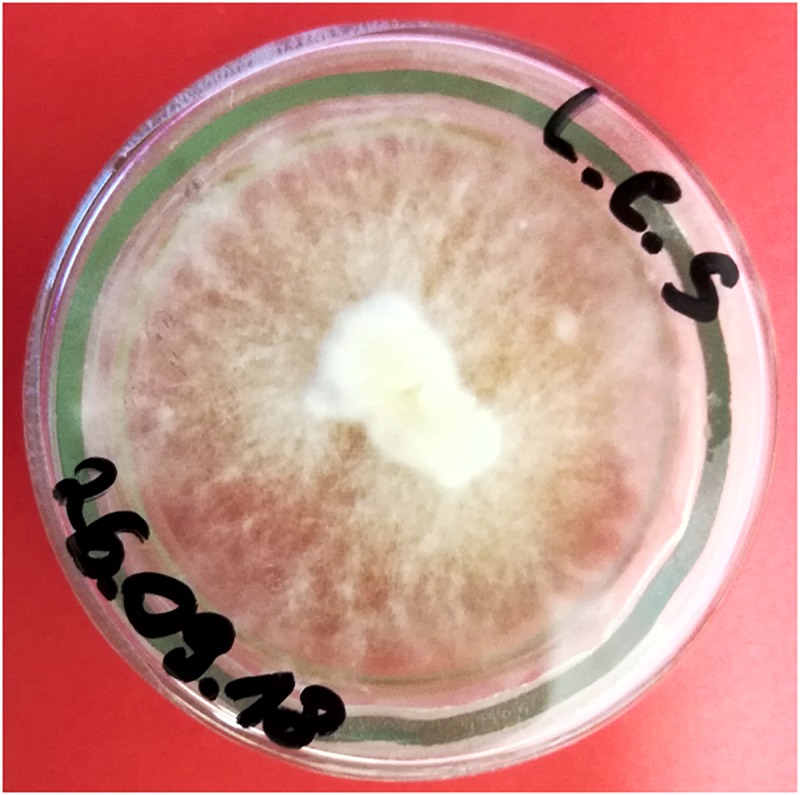
*Lentinula edodes in vitro* culture on solid medium (picture made by Bożena Muszyńska).

Cultures from the solid medium were used to establish experimental cultures cultivated on the modified liquid Oddoux medium (Oddoux, 1957). The initial inoculum from the solid medium was 0.1 g. The cultures were shaken at a rate of 140 rpm (ALTEL, Poland) and incubated at a temperature of 23 ± 2°C under a photoperiod (10-h light, 900 lx, and 14-h dark). The agitated liquid cultures of *L. edodes* were maintained for 2 weeks, and then subcultured.

### Experimental *in vitro* Culture

Mycelium of *L. edodes* was passaged to Erlenmayer flasks containing 250 mL of liquid Oddoux medium with addition of piroxicam in daily dosages used by human (10, 20, and 80 mg). The flasks were put into rotary shaker (ALTEL, Poland) with 140 RPM for 2 weeks in 23 ± 2°C under a photoperiod (10-h light, 900 lx, and 14-h dark). After 14 days of incubation, biomass was separated from the medium, rinsed with redistilled water and prepared for lyophilization as well as separated liquid medium. Then, the obtained mycelium from *in vitro* cultures and culture media were subjected to the lyophilization process (Freezone 4.5, Labconco) (Picture 2).

**PICTURE 2:**
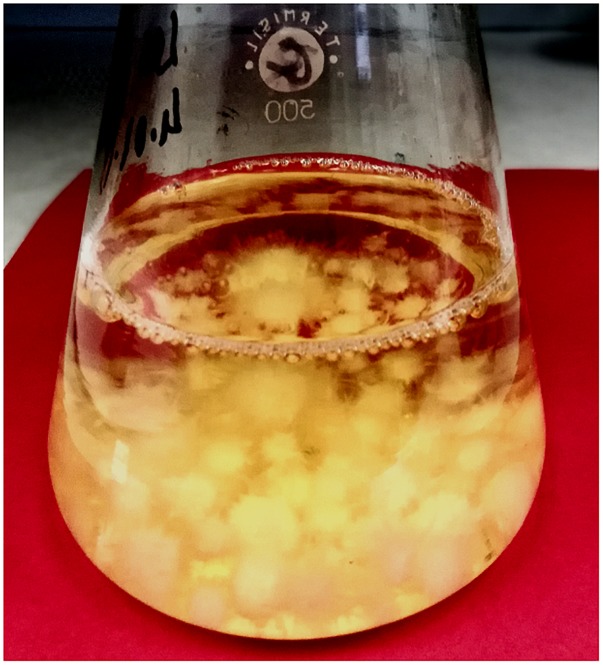
Experimental *in vitro* culture of *L. edodes* with addition of piroxicam in liquid Oddoux medium (picture made by Bożena Muszyńska).

### Sample Preparation

The powdered in a mortar mushroom materials (mycelium from *in vitro* cultures on media containing piroxicam and culture media) at 5 g were extracted with a mixture of methanol and dichloromethane in ratio: 75:25 (*v/v*) in an ultrasonic bath at 49 kHz for 30 min (Sonic-2, Polsonic). Merged extracts (300 mL) were concentrated to dryness using a rotary vacuum evaporator at 22 ± 2°C, and were subjected to UPLC/MS/MS analysis.

### UPLC/MS/MS Analysis

The UPLC-MS/MS system consisted of a Waters ACQUITY^®^ UPLC^®^ (Waters Corp., Milford, CT, United States) coupled to a Waters TQD mass spectrometer (electrospray ionization mode ESI-tandem quadrupole). Chromatographic separations were carried out using the Acquity UPLC BEH C_18_ column (2.1 × 100 mm, 1.7 μm particle size), equipped with Acquity UPLC BEH C_18_ VanGuard pre-column (2.1 × 5 mm, 1.7 μm particle size). The column was maintained at 40°C, and eluted under gradient conditions using from 95 to 0% of eluent A over 10 min; a flow rate of 0.3 mL min^-1^. Eluent A: water/formic acid (0.1%, *v/v*); eluent B: acetonitrile/formic acid (0.1%, *v/v*). Chromatograms were recorded using Waters eddd PDA detector. Spectra were analyzed in 200–700 nm range with 1.2 nm resolution and sampling rate 20 points s^-1^. MS detection settings of Waters TQD mass spectrometer were: source temperature 150°C, desolvation temperature 350°C, desolvation gas flow rate 600 L h^-1^, cone gas flow 100 L h^-1^, capillary potential 3.00 kV, cone potential 30 V. Nitrogen was used for both nebulizing and drying gas. The data were obtained in a scan mode ranging from 50 to 1000 *m*/*z* in time 0.5 s intervals; 8 scans were summed up to get the final spectrum. Collision activated dissociations analyses were carried out with the energy of 40 eV. Consequently, the ion spectra were obtained by scanning from 50 to 500 *m*/*z* range. Data acquisition software was MassLynx V 4.1 (Waters).

## Results and Discussion

A variety of methods were used to remove drug contaminants from wastewater and drinking water treatment, such as sonochemical degradation of ibuprofen (Xiao et al., 2014), naproxen (Im et al., 2013), diclofenac (Naddeo et al., 2013). Other ways to removing possible pollutants are phytoremediation or mycoremediation (Nowrotek et al., 2016; Topal et al., 2016; Rodríguez-Espinosa et al., 2018).

Researches about usage of WRF for wastewater treatment have been conducting for many years (Pointing, 2001; Lamrood and Ralegankar, 2013). The ability of the WRF to degrade various pharmaceutical compounds, which can be transmitted to the environment and thus be potentially harmful, has also been reported (Tsujiyama et al., 2013; Riggins and Gregory, 2015; Eldridge et al., 2017; Muszyńska et al., 2018b).

Based on research data there are some works about using fungi in the process of decomposition of polycyclic NSAIDs (Suryanarayanan et al., 2012; Domaradzka et al., 2015). Complete degradation was described only for olsazine (Razo-Flores et al., 1997). The first step of the transformation is most often hydroxylation catalyzed by cytochrom P-450 monooxygenases, or oxygenation by laccases and three peroxidases: lignin peroxidase, manganese-dependent peroxidase and versatile peroxidase manganese-dependent peroxidase. Applied species were able to dechlorinate halogenated aromatics (Marco-Urrea et al., 2009). Gonda et al. (2016) presented the first research about fungal transformation of piroxicam and diflunisal. These authors discussed the ability of the species used to biotransformation of chosen medicines, and the possibility of using high concentrations of these substances and their chemical similarity. Compounds produced by fungi (e.g., *Aspergillus nidulans*, *Bipolaris tetramera*) were similar to metabolites, which can be less or more toxic compared to the original substances. During oxidation the polarity of compounds increases, which may affect their less bioaccumulation. However, it may happen that thanks to fungal enzymes, this process can be increased. From the investigated drugs, piroxicam was the most resistant to used strains (Gonda et al., 2016).

Jimenez et al. (2018) described biological, photochemical and thermal degradation of three oxicams in samples river water. Obtained results indicated that the direct sunlight irradiation promoted a fast degradation of drugs, while the chemical reactions in solution were less important. After about month several degradation products were found. Authors proposed their structures and pathways of degradation, created by photochemical reactions as the main reason of the degradation of the oxicams in water, except hydrolysis.

Continuing the implementation of the subject our team (Dąbrowska et al., 2018) made research project about the ability of *L. edodes*’s mycelium from *in vitro* cultures to degrade antibiotics. Authors showed that the examined mycelia take short time duration to remove the cefuroxime axetil from the medium. An observed mycoremediation process can be used as an alternative to other methods for remediation cephalosporins contamination.

In presented experiment good biomass growth for *L. edodes* could be obtained from agitating liquid cultures on the modified Oddoux medium and on the same medium with addition of piroxicam. The dynamics of mycelium growth in liquid Oddoux medium did not differ from that registered in earlier researches, and with addition of piroxicam was in the same level (Muszyńska et al., 2015, 2018a). The addition of piroxicam to medium lead to increase of *L. edodes* biomass in their culture *in vitro* (average to 1.2 g dry weight per 1 L of mushroom medium). However, growth stimulation was not dependent on applied dose of piroxicam.

**FIGURE 1:**
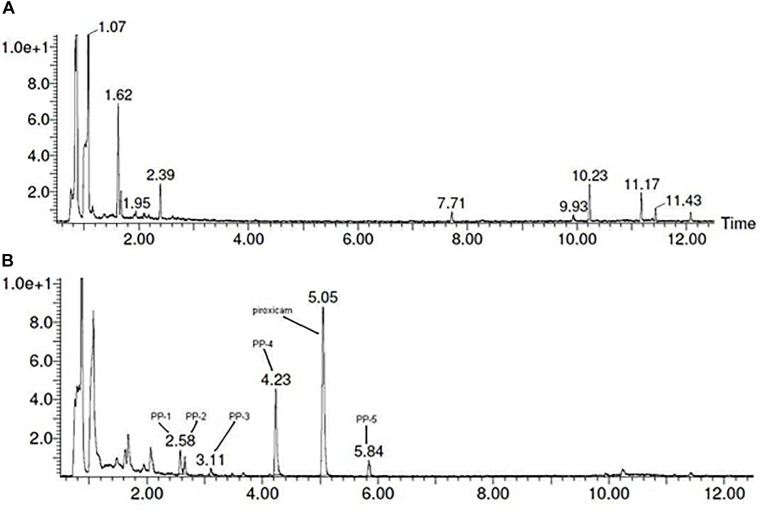
UPLC-DAD chromatograms obtained for: **(A)** Oddoux medium from *in vitro* culture of *L. edodes*; **(B)** Oddoux medium from *in vitro* culture of *L. edodes* with addition of piroxicam (20 mg/250 mL) in the beginning of logarithmic phase of mycelium growth.

Analysis of the extracts of the medium and mushroom mycelium with no addition of piroxicam showed no peaks on UV chromatogram thus all the compounds observed on the chromatograms of the extracts of the mushroom materials with added drug were most probably products of its biodegradation (Figure 1).

**FIGURE 2:**
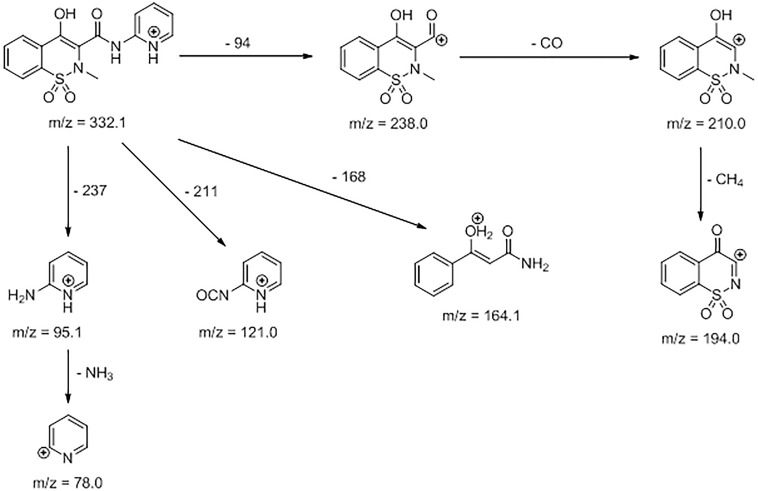
Proposed fragmentation pattern of piroxicam by mycelium from *in vitro* cultures of *L. edodes*.

The identification of the degradation products of piroxicam was performed on a basis of UPLC/MS analysis and supported with fragmentation patterns obtained from MS/MS experiments. The proposed structures of the degradation products are shown in Table 1. The proposed fragmentation patterns of piroxicam and its degradation products are shown on Figure 2.

**Table 1:** Proposed structures and fragmentation pattern of piroxicam by mycelium from *in vitro* cultures of *Lentinula edodes*.

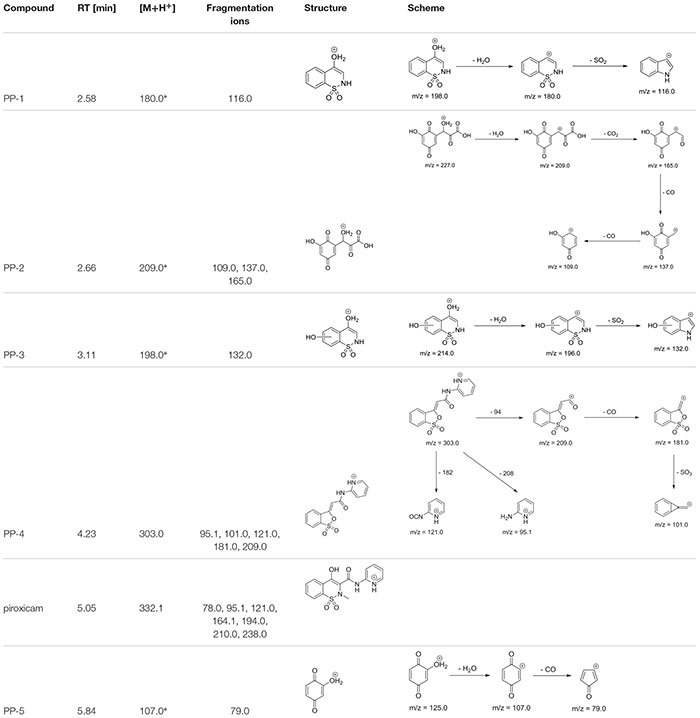

*^∗^[M–H_2_O]^+^*

The degradation process was found to affect primarily the linker between the thiazine and the piperidine ring, leading to its oxidation and cleavage. Additionally, oxidation of the benzothiazine moiety was observed, leading to hydroxylation and oxidation of the phenyl ring (PP-2, PP-3, and PP-5), as well as oxidation of the thiazine ring leading to partial (PP-4) or complete removal (PP-2 and PP-5) of the sulfonamide moiety (Table 1). The molecular ion ([M+H^+^]) peak of *m/z* 332.1 fragmented into 7 ions of *m/z* 78.0, 95.1, 121.0, 164.1, 194.0, 210.0, 238.0. Based on the fragmentation ions for obtained degradation products (PP-1 to PP-5) and the available literature data, we have attempted to identify possible ways of degradation of piroxicam in fungal environment. It seems that the degradation process led finally to 2-hydroxybenozquinone, which may be further oxidized yielding inorganic compounds. What more piroxicam biodegradation was on the same level for used different concentration of piroxicam (10; 20; 80 mg per 250 mL of medium), what may suggest, that the ability of the mushroom to degrade the drug was not exceeded even in the highest concentration of piroxicam.

The presence of electron withdrawing functional groups, such as amide, carboxylic, halogen, and nitro group generates an electron deficiency and thus renders the compounds less susceptible to oxidative catabolism. Whereas while electron-donating functional groups, such as amine, hydroxyl, alkoxy, make the molecules more susceptible to electrophilic attack by oxygenases (Yang et al., 2013). In this review authors reported many cases of bioremediation for drugs form various classes, quoting source works. To our best knowledge such studies for piroxicam have not been carried out yet. A specific class of enzymes, commonly secreted by WRF, called LMEs, is of particular interest for biodegradation of pharmaceuticals. The most studied LMEs are glycosylated heme-containing peroxidases, lignin and manganese peroxidases, and a copper-containing phenoloxidase and laccase (Pointing, 2001; Janusz et al., 2017). LMEs are primarily oxidoreductases that catalyze the flow of electrons from one substrate to another, and work by generating free radicals that randomly react with the lignin polymer, breaking covalent bonds and releasing phenolic compounds. Thanks to such mechanism, advanced oxidation reactions take place. The lignin degrading-system is extracellular, non-stereoselective and non-specific, able to cleave the carbon-carbon and carbon-oxygen bonds, regardless of the conformation of the chiral carbon of lignin (Fernando and Aust, 1994). This feature is partly due to highly reactive free radical degradation mechanisms that are ideal for the biodegradation of organic pollutants in the environment. Gonda et al. (2016) described a degradation pathway of piroxicam based on fragmentation pattern developed by other authors. Presented fragmentation showed, that the pyridine ring was hydroxylated, and a new compound was identified as hydroxypiroxicam.

The results obtained in presented research, are in accordance with this observation, since pyridine and thiazine moieties, which are electron rich regions of the piroxicam molecule, were readily degraded. At the same time phenyl ring was found to be quite stable and underwent only partial degradation *via* oxidation to 2-hydroxyquinone.

Results received under described work indicate on a high level of the degradation degree of examined drug by analyzed mycelia. These information shows necessity for further studies, which are still indispensable in order to have a comprehensive knowledge of ability of WRF to remediate pharmaceuticals.

## Conclusion

Pharmaceuticals in the environment can potentially cause to the formation of resistant strains of bacteria as well as recalcitrant toxins. Presented pilot tests showed that the examined mycelium from *in vitro* cultures of *L. edodes* mushroom take short time duration to remove the piroxicam from the medium, and thus mycoremediation can be used as an alternative to other methods for remediating contamination of this compound. Thanks to reproducible culture conditions, mycelium from *in vitro* cultures used in the experiment allow the monitoring of the accumulation and biodegradation of piroxicam from the media and the determination of the degree of bioaccumulation, and this further allows the selection of species for environmental remediation from this compound which are detrimental to living organisms. The results confirm the possibility of the use of the investigated mycelia of *L. edodes* from *in vitro* cultures, in remediation of piroxicam.

## Author Contributions

BM and WO conceived and designed the study. BM performed the experimental section. MD and MS cooperated in conducting the experiments, and wrote and revised the manuscript. JL and JP-P performed the linguistic correction. PŻ performed the UPLC/MS/MS analysis.

## Conflict of Interest Statement

The authors declare that the research was conducted in the absence of any commercial or financial relationships that could be construed as a potential conflict of interest.

## Footnotes

1 http://www.mycokey.com
